# Water-Based Aerosol for Book Deacidification: Experimental Apparatus and Theoretical Interpretation of Results

**DOI:** 10.3390/molecules26144249

**Published:** 2021-07-13

**Authors:** Giuseppe Chidichimo, Alessandra Crispini, Antonio Tursi, Maria Rita Basile, Ilaria Lania, Giovanni De Filpo, Cesare Oliviero Rossi, Francesca Scarpelli

**Affiliations:** Department of Chemistry and Chemical Technologies, University of Calabria, 87036 Rende, Italy; giuseppe.chidichimo@unical.it (G.C.); alessandra.crispini@unical.it (A.C.); basimari88@gmail.com (M.R.B.); ilarialania.il@gmail.com (I.L.); giovanni.defilpo@unical.it (G.D.F.); cesare.oliviero@unical.it (C.O.R.); francesca.scarpelli@unical.it (F.S.)

**Keywords:** paper deacidification, pH, X-ray fluorescence, FTIR spectroscopy, PXRD, alkaline reserve

## Abstract

One of the major problems in book conservation is the long-term deconstructive effect of acidity introduced into the paper by several additives, which, in the presence of humidity, generates a hydrogen cation with a strong catalytic role in cellulose depolymerization. Many types of treatment have been used in the past, but up to now, research for less-invasive, fast and cheap methods is still vividly ongoing. In this study, an approach to book deacidification is presented, where alkaline water solutions are administered to bound books in the form of micrometer-sized aerosol droplets, without using vacuum apparatus accessories. Alkaline clouds treatments were alternated with gentle air fluxes of drying steps. Few cycles are required to achieve uniform deacidification of books. The treatment could be conducted with proper apparatus on large volumes, resulting in rapid treatment time and low cost. The titration curve reporting the variation of book pH, with respect to the amount of absorbed alkaline aerosol, was built and interpreted in terms of a chemical model for the neutralization process. FTIR, PXRD and XRF spectroscopies were used to characterize the book chemistry. The effects of the treatment on the book were evaluated by measuring the degree of polymerization (DP) of the paper and the colorimetric coordinates of the paper and ink. Artificial aging tests revealed a general increase in the aging stability of the deacidified paper samples with respect to the untreated samples. Finally, the alkaline reserve data are discussed.

## 1. Introduction

The mass deacidification of paper works and library archives is currently a problem of considerable importance. Every year, tens of tons of books are treated in order to facilitate their conservation and usability and improve the appearance of the paper [1,2].

Millions of tons of acid paper have been produced over the past two centuries, and acid paper is still mainly produced in Asia. It is estimated that 30% of Indian publications and about 5–10% of paper in Europe and the United States require deacidification processes. Several studies have evaluated the consequences of the hydrolysis and oxidation processes of hundreds of millions of bound and unbound paper documents in different collections around the world [2,3]. Cunha (1987) [4] estimated that a typical library loses about 5% of the value of books each year, while Smith (1987) [5] calculated that the Library of Congress has losses of around USD 200,000 each day due to hydrolysis processes occurring during book storage. 

**Figure 1:**
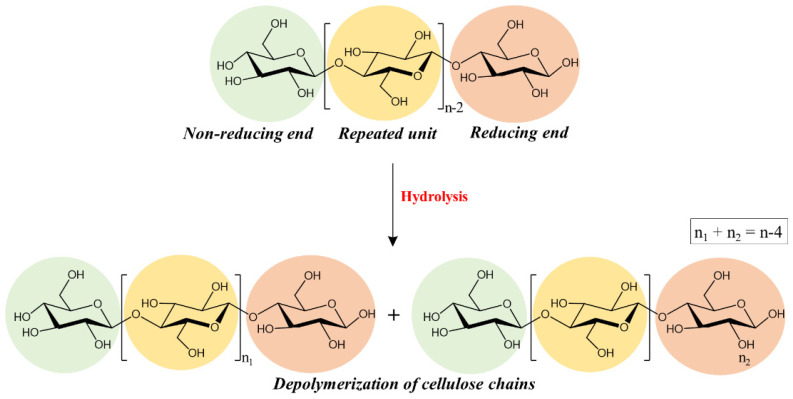
Hydrolysis of cellulose.

The raw material usually used for the production of paper is cellulose, the most abundant organic polymer in nature, formed by glucose monomers organized in chains and linked together by hydrogen bonds [6,7]. Several past paper production techniques (especially those from the 19th century with the addition of alum rosin sizing and industrial paper production from wood) introduced conservation problems related to its acidity, including the acid-catalyzed depolymerization of cellulose and oxidative processes [8,9]. In particular, ancient paper artifacts have high acidity from their production due to the many substances added during the production process (alum, metal ions, rosin and additives) [1,10]. The presence of these substances and high levels of relative humidity dramatically promotes the acid hydrolysis of cellulose [11]. The bonds between the different glucose units (1,4 glycosidic bond of cellulose) are broken due to the insertion of water molecules, leading to the cleavage of the glycosidic bond (which results in a decrease in the degree of polymerization of cellulose), as illustrated in Figure 1 [1,12]. In addition to acid hydrolysis processes, oxidation reactions take place at the reducing end of polymer chains in the C1, C2 and C3 positions of the glycosidic ring generating acid species [13,14,15].

Concerning book preservation, an important common goal is to neutralize the acids already present in the paper and introduce an alkaline reserve to protect books from the possible formation of further acidic compounds during natural aging. In order to inhibit the acid-catalyzed cleavage reactions of the cellulose chains of the paper, the use of alkaline compounds allows neutralizing the protons (H^+^) responsible for the acidic pH. Currently, several conservation and restoration methods are employed in order to deacidify the paper of books [2,16]. The most commonly used deacidification treatments, also carried out on entire volumes, are divided into two classes: non-aqueous and aqueous processes. The main non-aqueous processes involve the use of carrier fluids such as fluorinated liquids or hexamethyldisiloxane in combination with alkoxides of alkali metals (non-aqueous solution) or fluorinated liquid or heptane as carrier fluids with MgO or other alkaline particles (non-aqueous dispersion) [2,17,18]. Some of the most accredited methods for deacidification, still in use and not, which can also be used for the treatment of entire volumes, are presented below:

1. The “Vienna process”, conceived in the 1970s, involves immersing the entire volume in vacuum in a solution of calcium hydroxide and methylcellulose as a consolidant [19]. The system is still used for the deacidification of newspapers [20];
2. The “Sablè” method is a French variation of the Wei T’o system invented by Richard D. Smith and developed by the National Archive in Canada. It was adopted between 1987 and 1989. The system consists of the insertion of open volumes, kept in a vertical position inside metal baskets, into an autoclave. The volumes are first dried at 45 °C and then impregnated for 10 min with a mixture consisting of ethoxy-magnesium carbonate and ethanol [21,22];
3. The Bookkeeper^®^ method, devised in 1985, is based on the use of magnesium oxide microparticles dispersed in perfluoroheptane and the addition of a surfactant. The volumes are placed on vertical supports and opened at an angle of 90° and then placed inside four reactors that operate sequentially and can treat 32 volumes in 2 h. The deacidifying product is inserted, after establishing vacuum conditions. This is one of the most popular methods for its speed of execution and relatively low costs. The Bookkeeper^®^ method provides a considerable reduction of paper degradation and does not affect inks and book ligatures. However, it is reported in the literature that this method appears less efficient for the treatment of large-sized books due to the fact that the alkaline reserve is low and the deacidification is not very uniform. The solvent seems to have a low penetration capacity into the paper fibers, and some white patina appears on the surface [2,21,23,24];
4. The “CSC Booksaver” method, developed in 2001, involves the use of n-propylated magnesium carbonate dispersed in heptafluoropentane. The volumes are first dehydrated and then impregnated with the deacidifying substance [20,25];
5. The “ZFB:2” method, devised in 2011, uses calcium carbonate and magnesium oxide dispersed in heptane. The volumes are not opened, and the process takes about four weeks [26];
6. The “Papersave” method from Battelle Ingenieurtechnik GmbH and the “Papersave Swiss” method use magnesium and titanium ethyloxides dissolved in hexa-dimethyl siloxane and provide good penetration and an alkaline reserve, reducing the degradation of cellulose with aging. However, the treatment cannot be applied to leather covers, pergamene covers, patinated papers and newspapers. Inconvenient decreases in paper mechanical strength, discoloration, white deposits and ink bleeding have been observed [27].

In general, it is asserted that the non-aqueous methodologies mentioned above have disadvantages and limitations such as the accumulation of by-products on the surface of the paper during the treatment process, the use of compounds that alter the appearance of the treated documents (non-aqueous treatments with magnesium compounds) and the use of solvents that are not environmentally friendly and/or that could also solubilize the inks, as well as the absence of the washing action of the water, which prevents the solubilized products from moving away [28,29,30]. Ultimately, all these methodologies require improvements to further facilitate the penetration of the deacidifying solution between the pages, increase the alkaline reserve and avoid the mentioned problems. In non-aqueous solvents, the alkaline medium remains at the state of micrometer-sized particles, and for this reason, they cannot easily penetrate into the paper fibers. In fact, there is evidence that they mostly remain on the external surfaces of paper sheets, rather than in the interior of the sheets [31], and that treatment applied to one side of paper by spray application does not affect the opposite sides [32]. On the other hand, the processes based on the use of aqueous solutions ensure good penetration of the solvent in the paper due to the hydrophilic character of cellulose. These should give, in principle, better spatial homogeneity and bigger alkaline reserves. Further, certain biocide protection should be given by the aqueous solution containing appropriate compounds, which, indeed, are very soluble in water [33].

One of the most used aqueous methodologies [34] consists of their complete disassembly and the immersion of single pages in two solutions: one of Ca(OH)_2_, which counteracts the acidity present, and one of Ca(HCO_3_), which deposits an alkaline reserve. The sheets are then left to dry, and the book is stitched back. However, this process is very expensive both in terms of time and money and allows treating only one book at a time [6,30,35].

Since the beginning of the 21st century, up to now, several patents [36,37,38,39,40,41] have appeared on book deacidification by means of a basic water solution fed to book pages in the form of aerosol droplets. These patented methods [36,37,38,39,40,41] make use of vacuum techniques at least in one of the process steps such as droplet injection or the subsequent drying of books. This condition introduces a certain degree of complexity to the treatment apparatus and represents a sort of limitation factor with respect to the diffusion of this interesting methodology.

## 2. Materials and Methods

### 2.1. Aerosol System

**Figure 2:**
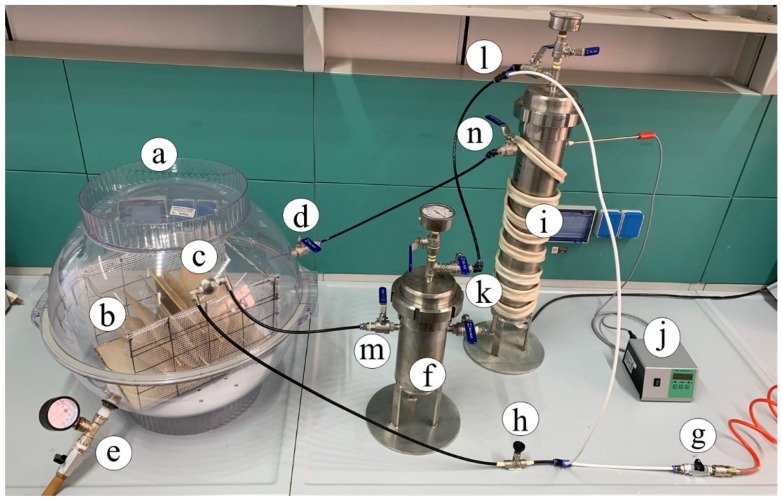
Deacidification homemade apparatus: (**a**) chamber hosting the book during treatment cycles; (**b**) book; (**c**) nuzzle nebulizer; (**d**) valve for dry air inlet; (**e**) valve for the exit of the drying air; (**f**) steel vessel alkaline solution container; (**g**) inlet compressed air; (**h**) T-valve connector; (**i**) steel desiccator; (**j**) temperature control device; (**k**–**n**) flux control valves.

The book deacidification experiments were performed by means of the homemade experimental apparatus shown in Figure 2.

**Figure 3:**
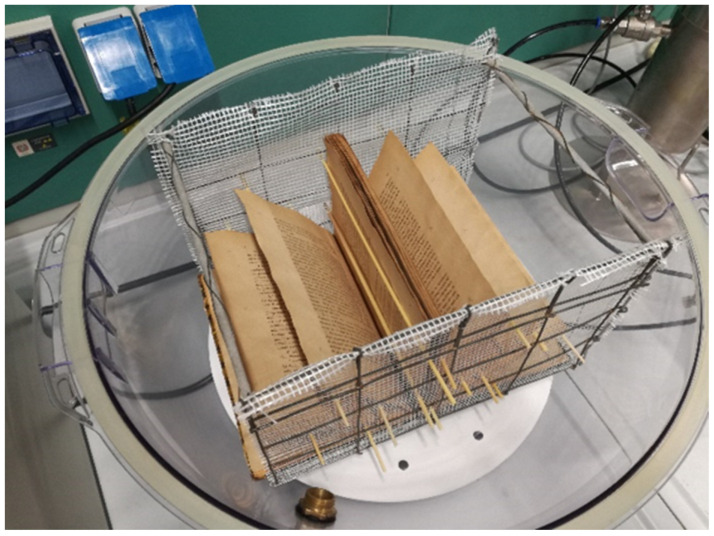
Details of the book holder grid with page spacers.

The chamber (Figure 2a) hosting the book (Figure 2b) during the deacidification treatment was a polypropylene flask made by two airtight hemispheric sectors overlapping along the equatorial circumference with a volume of 65 L. The book was suspended below the equatorial plane of the flask by means of an appropriate grid. An IKEUCHI BIMK6004 S303 nozzle nebulizer (TKK Corporation, Thailand) (Figure 2c), able to uniformly spread a basic solution cloud in the form of 20–30 µm droplets on the open book pages, was mounted on the superior hemisphere of the flask. On the same superior hemisphere of the flask, a connector carrying a valve for dry air inlet (Figure 2d) was placed. The bottom hemisphere was fitted with an additional valve (Figure 2e) to allow the exit of the drying air to the external environment and maintain the inner of the flax at room pressure. A Ca(OH)_2_ saturated water solution with a pH equal to 12.24 (molar concentration of Ca(OH)_2_ equal to 8.7 × 10^−3^ mol·L^−1^) was provided for deacidification means by using the steel vessel (Figure 2f). The basic solution was fed into the flask from the bottom of this container by pressurizing the surface of the solution at a pressure of 1.5 atm by means of an air compressor (not shown in the figure) connected through a valve (Figure 2g). The compressor, by means of the T-connector (Figure 2h), further fed the air required by the nozzle. Once a humidification stage of the book pages was completed, warm and dry air, coming from the steel desiccator (Figure 2i), filled with silica gel, and thermostated by the power supply and controller (Figure 2j), was passed through the flask (Figure 2a). During the drying stages, the bottom valve of the flask (Figure 2e) was kept open, and all valves (Figure 2h,k–n) were manually settled to cut the fluxes in and from the steel vessel (Figure 2f) and maintain the fluxes in and from desiccator (Figure 2i). In order to ensure a uniform spread of the nebulized solution, pages were maintained well and separated from each other by using a grid holder, where appropriate tin wood rods could be inserted as page spacers, as indicated in Figure 3.

**Figure 4:**
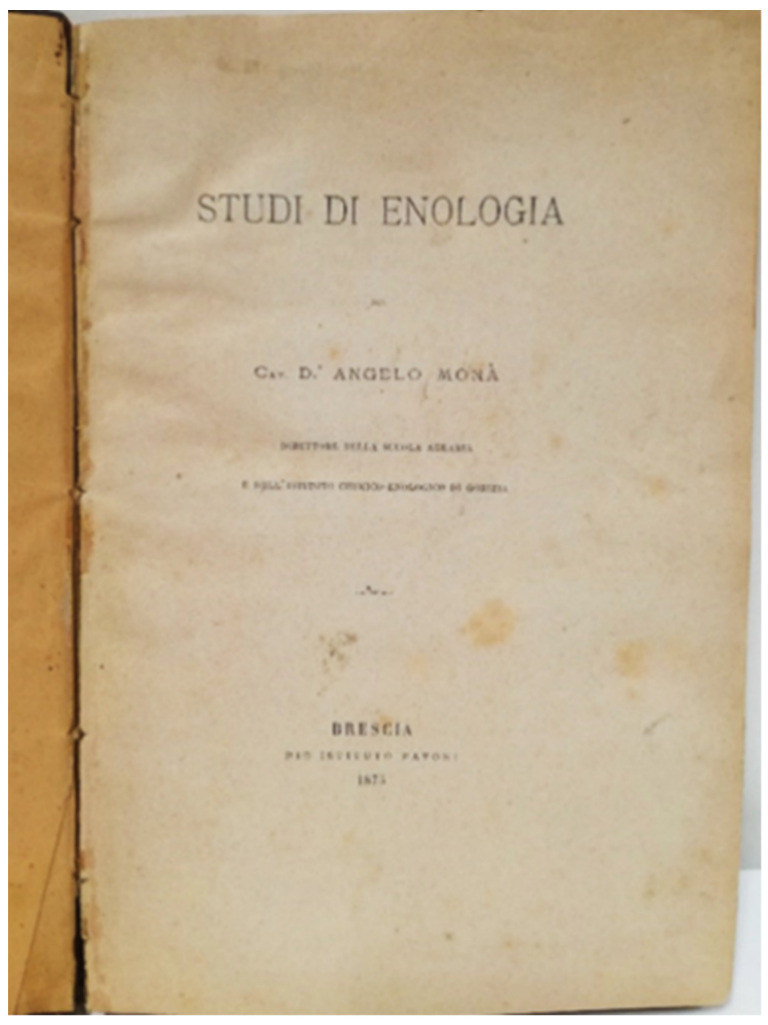
Photography of the title page of the analyzed book.

Deacidification experiments were performed according to the following steps:

1. The book “Studi di Enologia” del cav. Angelo Mona (Brescia—Pio Istituto Pavoni—1875), the title page of which is shown in Figure 4, after being weighed, was supported on the holder, as indicated in Figure 2b, and inserted into the flask. At the actual development stage of our homemade apparatus, only a limited number of pages could be homogeneously treated with the alkaline droplets. For this reason, only 12 (from 1 to 6 and from 91 to 96 pages) of the almost 200 pages of the book were kept wide open to facilitate a uniform deposition of the aerosol through a single nozzle. The other pages were kept firmly closed in order to exclude them from the deacidification process;
2. The book, placed into the flask, was exposed to a dry air flux at the temperature of 45 °C for 15 min. Then, it was extracted from the chamber and weighed. The weight variation was 3.45%;
3. The book was replaced in the chamber. A cloud of nebulized solution was fluxed above the open pages through the nozzle placed on the top hemisphere. The aerosol inlet was left to flow for 3 min, while the valve on the bottom of the flask was kept open to ensure a constant pressure in the flask;
4. The book was removed from the flask and weighed in order to measure the amount of the absorbed basic solution and inserted back into the treatment chamber;
5. Dry air at the temperature of 45 °C was let gently fluxed into the flask for 3 min, entering with the help of a small pressure gradient (around 0.05 atm) and exiting through the bottom open valve. The rehydration of the air gradually removed the absorbed humidity from the book. After the above dehydration cycle, the book was weighed. This step was repeated 5 times in order to bring the book to a constant weight;
6. The pH on every treated page was measured in six different points, as described in Section 2.4, and the average value was recorded;
7. The procedure described above, as defined in Steps 3 to 6, was repeated 8 times in order to reach a satisfactory pH value.

### 2.2. X-ray Fluorescence (XRF)

Analysis was performed by a Bruker Tracer III with an Rh/Pd X-ray source under the following conditions: voltage, 40 kV; current, 11 μA; acquisition time, 15 s. Measurements were taken in six different points of the pages in all 12 pages investigated. XRF data were evaluated by Artax software from Bruker. The software provides qualitative and partially semi-quantitative information with respect to the elements detected. These data represent the values of the integrals subtended by the characteristic peaks of the emitted fluorescence by the different detectable elements. They depend in principle on many factors, but when measurements are appropriately made, it can be assumed that XRF intensities are in the first approximation proportional to the density of the chemical elements dispersed into the sampled volume and to a specific emission constant, which is different for each of the elements. Thus, significant information on the chemistry of the book could be derived from XRF signals.

### 2.3. FTIR Spectroscopy

FTIR-ATR analysis was performed by using the FTIR ATR BRUKER ALPHA II spectrophotometer equipped with the accessory for attenuated total reflectance (ATR) with a diamond and crystal window. Analysis was carried out with a resolution of 4 cm^−1^ in the region between 4000 and 375 cm^−1^, setting 48 scans for a single analysis. The recorded spectra were interpreted using the Bruker OPUS software.

### 2.4. Powder X-ray Diffraction (PXRD)

PXRD patterns were acquired on a Bruker D2-Phaser equipped with Cu Kα radiation (λ = 1.5418 A) and a Lynxeye detector, at 30 kV and 10 mA, with a step size of 0.01° and a step time of 1.5 s (acquisition time: 11,250 s), over an angular range of 5–80° 2θ. A 1 cm^2^ area of the book was sacrificed to perform the PXRD analysis before and after deacidification treatment. Small pieces of paper sheets, as they were, were placed on a zero-background sample holder. Slight 2θ displacements of some reflection peaks, which did not exceed ±0.3°, with respect to the reference patterns reported in the Crystallography Open Database (COD) and in the RRUFF database, could be ascribed to both the sample preparation and the instrumental set-up.

### 2.5. Contact pH Meter

The surface pH method (TAPPI T 529 om99) [42] was used for the pH measurement. Measurements of pH in standard conditions were made by placing a polypropylene plastic sheet beneath the investigated page. On each of the measurement points, a 40 µg drop of MilliQ water (HANNA HI70960 conductive electrolyte solution) was added dropwise by a droplet-dosing cup bottle. The surface electrode was then set on the water droplet. Both these operations were performed in rapid succession in a time lower than 1 s. The pH was measured by a HANNA—Leather and Paper HI 99171 pH meter with a surface glass. Reported pH values are the mean values calculated from the pH measurements, taken from six test points, in each of the twelve pages investigated. 

### 2.6. Artificial Aging 

Artificial aging tests were performed according to the methods developed by the Conservation Processes and Materials Research Division of the Canadian Conservation Institute (CCI) in Ottawa (Canada) and the Preservation Research a Testing Office of the U.S. Library of Congress (LOC) in Washington (USA) [43]. Six strips of about 0.2 g, taken from all of the deacidified book pages (from 1 to 6 and from 91 to 96), and six analogous strips, cut from 41 to 46 and from 107 to 112 pages (taken out from the book before any treatment), were aged. All paper samples were conditioned at 23 °C and 50% RH for ≥24 h both immediately prior to and following the accelerated aging period. The preconditioned paper samples were inserted inside closed glass bottles with a proportion of paper weight/bottle volume of 1 g/36 mL and introduced into the heated oven at 100 °C for 120 h in one continuous period.

### 2.7. Colorimetry and Optical Microscopy

Colorimetric measurements CIE L*a*b* were performed to ascertain the effect of the deacidification treatments on paper and ink color before and after aging. The ARW-265 portable colorimeter (Arroweld Italia Spa, Italy) with a measurement caliber of 8 mm was used in the D65/10° (light source) mode. The paper colorimetric coordinates were measured, for all sample types (untreated: Pages 41, 44, 45, 107, 109, 112; deacidified: Pages 1, 4, 5, 92, 93 and 95; untreated and aged: corresponding aged strips of Pages 41, 44, 45, 107, 109 and 112; deacidified and aged: corresponding aged strips of Pages 1, 4, 5, 92, 93 and 95), on six different testing points, opportunely chosen on each of the above-mentioned book pages. In particular, the colorimetric ink investigations were performed, according to Sequeira et al. (2006) [1], on six “a” letters for each of the different page samples. The total color difference (ΔE*) and the whiteness parameter (W) were calculated according to Brainard (2003) [44] and Gooch (2011) [45], respectively. The averaged values and standard deviations were determined. The script character was observed by a Zeiss Telaval 31 inverted phase contrast optical microscope (Zeiss, Oberkochen, Germany) with an S5A-6V halogen lamp (25 W) as a light source in order to detect the presence of eventual bleeding effects.

### 2.8. Determination of the Degree of Polymerization (DP)

The polymerization degree for each of the different paper sample types, as in the case of the colorimetric measurement (untreated, deacidified, untreated and aged, deacidified and aged), was determined according to the UNI 8282 standard test [46] by measuring the viscosity limit of cellulose solutions in bis-(ethylenediamine)-copper (II) hydroxide (CED) 0.5 M. The CED solution was prepared by diluting the Aldrich^®^ 1M CED solution in water. A 10 mg amount of paper for each paper sample was dissolved in 20 mL of CED at 25 °C by stirring the system for at least 30 min. In order to obtain the required limit viscosities, 5 solutions for each of the investigated paper samples were analyzed: the base solutions, obtained as described above, and 4 solutions, obtained by diluting the base solution with 0.5 M CED. Four dilution ratios, 2, 4, 8 and 16, were used. Viscosity measurements were carried out at room temperature (25 °C) using an RFS III shear-strain-controlled rheometer (Rheometrics Co. Ltd., Piscataway, NJ, USA) equipped with parallel plate geometry (gap, 1.000 ± 0.001 mm; diameter, 50 mm) for the samples analyzed and a Peltier system (±0.1 °C) for temperature control. Steady flow experiments were performed, and the viscosity was measured at different shear rate values. The variation of viscosity versus shear rates for all samples was measured. It was observed that all samples had Newtonian rheological behaviors, which meant the viscosity was independent of the shear rates. Specific [η] viscosity was obtained according to Equation (1):

(1) $$\left[\eta \right]=\underset{c\to 0}{\lim}\frac{\eta_{\mathrm{sp}}}{c}$$

where c is the solution concentration, and ηsp = (η − ηs)/ηs, where ηs is the viscosity of the CED solvent by determining the vertical intercept of the ηsp plot versus the concentration. Finally, the degree of polymerization (DP) was calculated according to the equation: DP = 1.33[η]^0.905^, according to UNI 8282:1994 [46]. Mean values, obtained by averaging those measured from Pieces 41/42, 43/44, 45/46, 107/108, 109/110 and 111/112 of untreated and untreated-aged book pages and those measured from Pieces 1/2, 3/4, 5/6, 91/92, 93/94 and 95/96 of deacidified and deacidified-aged book pages, were reported.

### 2.9. Alkaline Reserve Determination

The alkaline reserve was determined according to the TAPPI 533 pm-92 method [47]. A 1 g amount of dry small paper pieces, taken in equal amounts from the untreated book pages and analogous quantities of paper taken from the deacidified pages, were used to investigate the original (before treatment) and final (after treatment) alkaline reserve, respectively. Each gram of the two of these paper samples was inserted into 25 mL of distilled water. A 20 mL volume of HCl 0.1 N solution was added to the suspension, which was then left for about 1 min at boiling temperature. After cooling, the suspension was titrated with a NaOH 0.1 N solution monitoring the color change of the indicator (close to pH = 7) previously added.

## 3. Results and Discussion 

Before discussing the deacidification process and the related model, FTIR, PXRD and XRF data will be presented in order to obtain a useful indication of the book chemistry. Spectral data were taken from the pages of the book kept open during the acidification process before and at the end of the deacidification treatment. pH values were measured, on the same pages, before the treatment and after each of the eight used aerosol/drying cycles. After discussing the deacidification process data, the effect of treatment on the degree of polymerization (DP) of the paper and the colorimetric coordinates of paper and ink, the variation of these parameters with aging and, finally, the alkaline reserve data will be discussed. 

### 3.1. FTIR Results 

**Figure 5:**
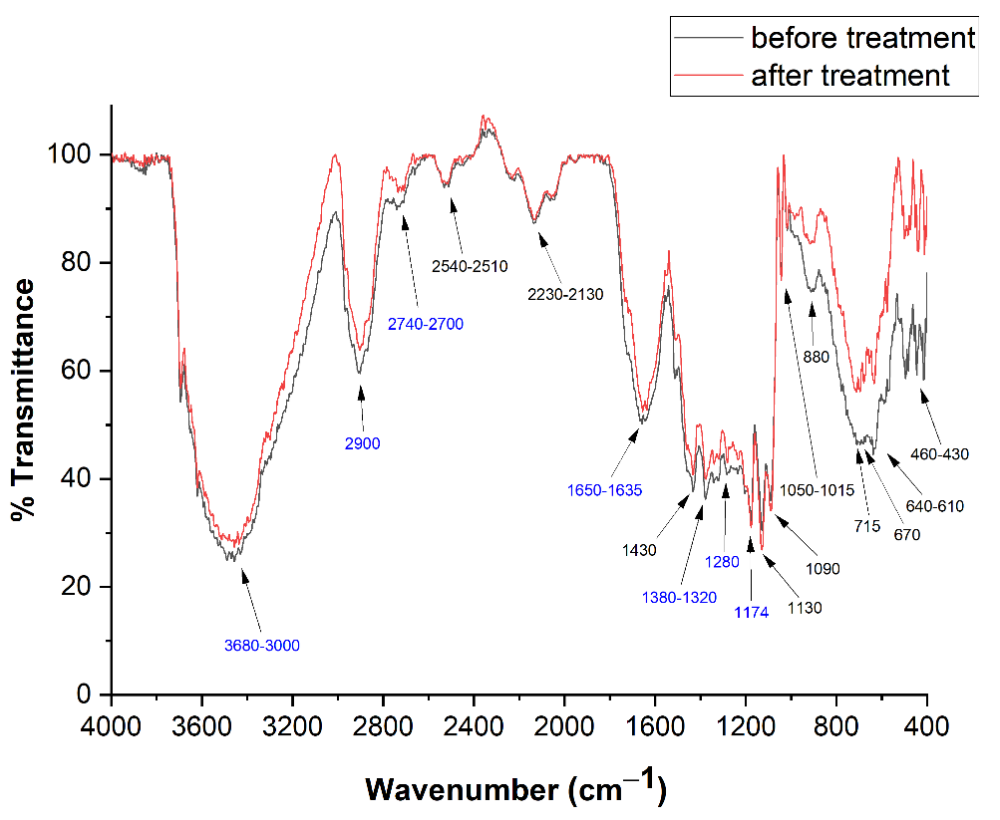
FTIR spectra before (black) and after (red) deacidification treatment recorded from page 1. Frequency values in blue are identified as cellulose peaks.

FTIR spectra are substantially equal for all the 12 pages investigated. Two copies of these spectra, precisely those recorded from Page 1, in pre- and post-treatment conditions, are reported in Figure 5, together with the transition wavenumbers. 

Those related to cellulose are reported in blue [48,49,50]. The identification of vibrational bands is reported in Table 1. 

An intense band, between 3680 and 3000 cm^−1^, is related to the OH stretchings of cellulose [46] and absorbed water, while the bending of this species appears at 1635 cm^−1^ [48]. The intensity of these bands is, of course, related with the degree of the paper hydration.

Cellulose CH stretchings appear between 2900 and 2740–2700 cm^−1^ [48], while CH bendings are at 1372, 1340 and 1320 cm^−1^ [49]; CH_2_ twistings and C=O stretchings are, respectively, at 1280 and 1735 cm^−1^ [48,49]. The band between 610 and 640 cm^−1^ can be attributed to Ca-OH bending [1]. On the other hand, spectra show the presence of calcium sulfate (CaSO_4_·2H_2_O) and calcium carbonate (CaCO_3_), the bands of which fall at 1130, 1050–1015 and 670 cm^−1^ [50] and 2510–2540, 2230–2130, 1430, 870–880, 715 and 460–430 cm^−1^ [51], respectively. These substances were used as fillers of the paper to improve its mechanical properties. Ultimately, FTIR spectra shown in Figure 5, taken before and after treatment, do not show appreciable variation, confirming that our treatment does not introduce a valuable modification in the number of significant functional groups (i.e., hydroxyls and carbonyls).

### 3.2. PXRD Results

**Figure 6:**
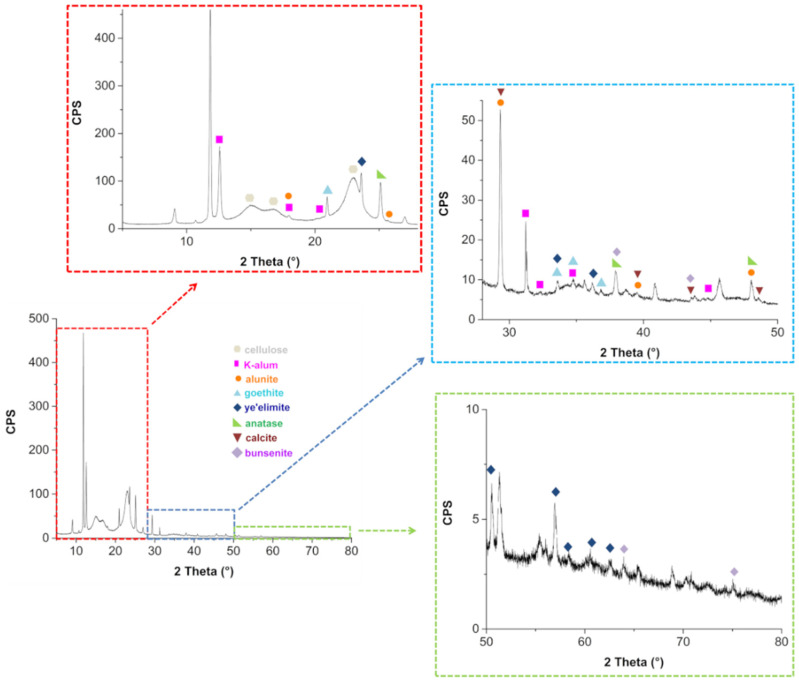
Untreated paper PXRD and related magnifications in the angular ranges 5–28° (red inset), 28–50° (blue inset) and 50–80° (green inset).

The untreated paper PXRD pattern is reported in Figure 6. The distinctive diffraction peaks of cellulose are visible at 2θ = 15.1°, 16.8° and 22.9° [52]. Several other reflections can be observed in Figure 6 (see insets), proving the presence of other crystalline species. 

These compounds were identified by comparison with reference patterns reported in the Crystallography Open Database (COD) and in the RRUFF database (Table 2). 

Finally, no differences between the diffractograms obtained after deacidification of the book were observed with respect to those obtained before the treatment. The presence of K-alum and alunite confirms the historical tradition of paper manufacturing of adding K-alum and other similar compounds into the ligno-cellulosic paste, together with animal gelatin [53,54]. While FTIR spectra show evidence of the presence of calcium sulfate bi-hydrate and calcium hydroxide, PXRD spectra do not show evidence of these compounds, at least in the crystalline phase. This is an interesting point that requires a deeper investigation, which is beyond the aim of this work. At the moment, we can only hypothesize that both calcium hydroxide and calcium sulfate may exist in the system as amorphous material dispersed on the cellulose surface. It is worth mentioning that iron appears to be present only as crystalline goethite. It is known [54] that epta-hydrate ferrous sulfate, traditionally defined as green vitriol, was often added to alum to partially substitute this much costly component. Further, ferric alum, a less pure form of alum, obtained by the sulfuric acid treatment of natural bauxite raw materials, could also have been used by paper manufacturing to replace part of alum. Goethite could be derived from bauxite, as this mineral represents one of the major sources of iron in bauxite [55]. Finally, no differences between the diffractograms obtained after deacidification of the book were observed with respect to those obtained before the treatment.

### 3.3. X-ray Fluorescence Results

**Figure 7:**
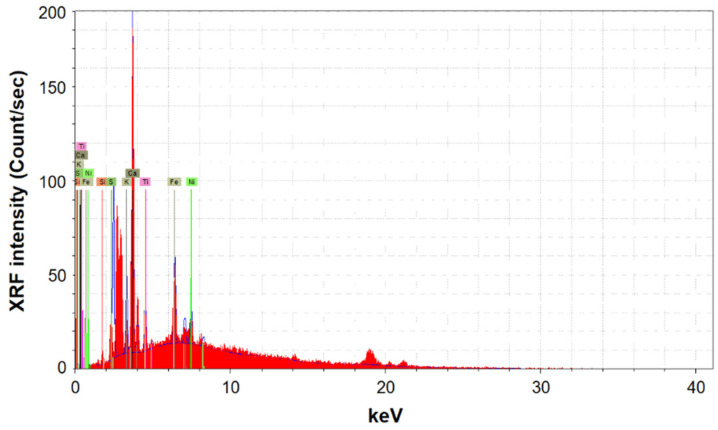
XRF spectra after treatment of Page 1.

Six spectra were recorded for each of the analyzed pages. Spectra were all similar to those reported in Figure 7. 

**Figure 8:**
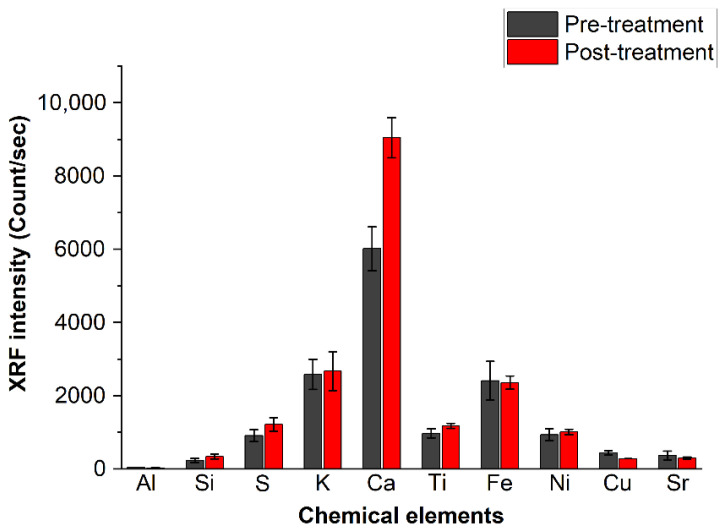
Intensity of the chemical species detected in the paper through XRF before and after deacidifying treatment. The error bars are the standard deviation as obtained from all measurements.

The averaged XRF intensity of each of the detected chemical elements, expressed in the scale applied by the instrument (count/s), is summarized in Figure 8. The average was taken on all data recorded from the 12 analyzed pages. The bars represent the standard deviations.

The elements detected through XRF analysis are Al, Si, S, K, Ca, Ti, Fe, Ni, Cu and Sr, from which those found with the highest intensity are calcium, iron, potassium and sulfur, confirming the findings obtained by FTIR and PXRD spectroscopies. The low aluminum intensity in the XRF spectra can be attributed to the low sensitivity of this element in the XRF technique. The XRF signal in Figure 8 shows, as expected from the beginning, that calcium is the only element increased by the deacidification process; the differences before and after treatment are far from the experimental error. 

**Figure 9:**
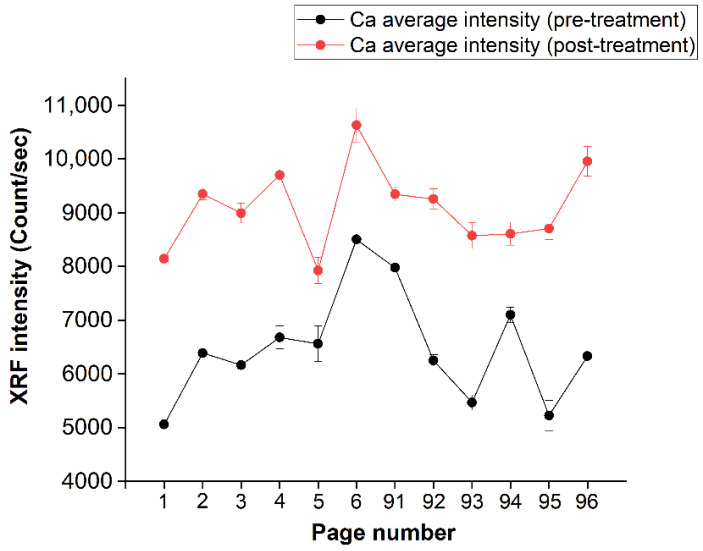
Average of the intensity values, per page, of Ca (before and after treatment). The pages are shown on the abscissa axis, while the intensity is shown on the ordinate axis.

In order to show the uniformity level of calcium deposition in the several analyzed pages, the intensity of the calcium “seen” by the XRF spectra for all pages is reported in Figure 9, where the black line represents the pre-treatment-averaged (on the page) calcium spectral intensity, while the red line shows the trend of the calcium signal after the treatment. An average difference of the order of about 50% can be observed.

### 3.4. pH Results

**Figure 10:**
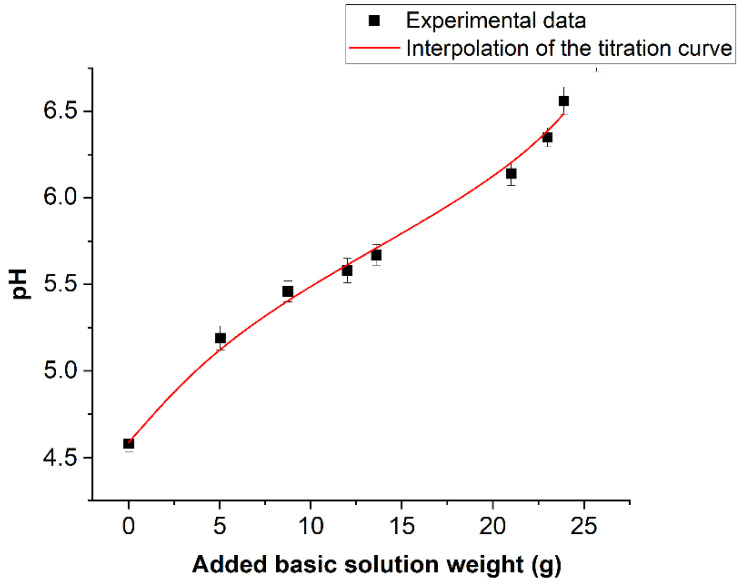
Trend of the average pH value measured after each complete deacidification treatment.

Figure 10 reports the values of averaged pH values measured on the book pages before treatment and after each nebulization/drying cycle. pH was also measured even on the book pages kept closed during the deacidification treatment. 

No pH variation was observed, confirming that the nebulized solution did not penetrate on these pages. On the abscissa axis, the total amount of basic solution absorbed by the book at the end of every cycle is reported. This quantity was calculated by the following Equation (2):

(2) $$W_{J}=\overset{J}{\underset{i=1}{\sum }}\Delta P_{i}$$

where ΔP_i_ is the variation of the book weight between the i-esimo nebulization stage and the (i − 1)-esimo drying stage. The hypothesis that W_J_ is proportional to the average calcium concentration added to the book at the J-esimo nebulization stage will be used in the following. If this increment of the calcium concentration is defined as b_J_, then the proposed hypothesis can be expressed by the equation b_J_ = βW_J_. The trend of the pH values against W_J_ can be considered as a titration curve of the book pages. The continuous line is the interpolation of the titration curve by the simple chemical model illustrated in the following. Taking into consideration the spectroscopic results shown above, it can be hypothesized that the acidity of the book is linked to the hydrolysis of some acid cationic species. To simplify the problem, the dominating effect of a single species was taken into consideration. The identification of this acid species will be discussed next, taking into consideration the acid constant of the involved hydrolysis process coming from the titration curve, as well as the information obtained from spectroscopic and X-ray diffraction data. The starting point is then the general assumption of the presence of a water-soluble compound C_n_A_m_, where C is hypothesized to be the cation species responsible for the book acidity, while A is its anionic counterpart (A could indeed correspond to more than a single anionic species, but this aspect would not affect at all the model). The presence of humidity leads to the electrolytic dissociation of C_n_A_m_, according to Equation (3):

(3) $$C_{m}A_{n}\;\to {\;\mathrm{mC}}^{n+}+{\;\mathrm{nA}}^{m-}$$

The acidity will then come out as a product of H^+^ due to the hydrolysis of the cation (4):

(4) $$C^{n+}+{\;H}_{2}O\;⇆C{\left(\mathrm{OH}\right)}^{p+}+{\;H}^{+}$$

where, for brevity, the superscript p^+^ was used instead of (n − 1)^+^, and K is the acidity constant of the hydrolysis process. The hydrolysis equilibrium is represented by Equation (5):

(5) $$\frac{\left[H^{+}\right]\left[C{\left(\mathrm{OH}\right)}^{p+}\right]}{\left[C^{n+}\right]}=K$$

In order to obtain a useful interpolating equation of the pH trend reported in Figure 10, [C(OH)^p+^] and [C^n+^] need to be expressed in terms of the C_n_A_m_ analytic concentration and in terms of the added Ca^2+^ during the book treatment and H^+^ concentrations. This can be achieved by using the following mass (6) and charge balances (7) at any J stage of treatment.

(6) $$\left[C^{n+}\right]+\left[C{\left(\mathrm{OH}\right)}^{p+}\right]=\;\mathrm{ma}$$

(7) $$\frac{n}{m}\left(\left[C^{n+}\right]+\left[C{\left(\mathrm{OH}\right)}^{p+}\right]\right)=\left[A^{m-}\right]$$

where a is the C_n_A_m_ concentration in the book. The presence of the OH^-^ concentration into Equation (8) leads to a third-order equation in H^+^, which complicates the finding of an analytic interpolation equation. For this reason, this term has been neglected.

(8) $$\left[H_{J}^{+}\right]+2b_{J}+n\left[C^{n+}\right]+\left(n-1\right)\left[C{\left(\mathrm{OH}\right)}^{p+}\right]=m\left[A^{m-}\right]+\left[{\mathrm{OH}}_{J}^{-}\right]$$

The model in this case must be considered strictly valid only when the OH^-^ concentration is two orders of degree lower with respect to the H^+^ concentration and only for the first five points of the graph reported in Figure 10. For the last three points, a greater approximation error of the order of 5% is introduced. However, such an approximation is of the order of the experimental error. The introduced simplification leads to the following second-order equation in H^+^ (9).

(9) $${\left[H_{J}^{+}\right]}^{2}+\left(2b_{J}+K\right)\left[H_{J}^{+}\right]+\left(2{\mathrm{Kb}}_{J}-\mathrm{Kma}\right)=0$$

Maintaining that b_J_ = βW_J_, with being W_J_ the amount of basic solution added to the book at the J-esimo nebulization treatment cycle, Equation (10) can be rewritten as Equation (11):

(10) $${\mathrm{pH}}_{J}=\log2-\log\;[\sqrt{{\left(2b_{J}-K\right)}^{2}+4\mathrm{Kma}}-\left(2b_{J}+K\right)]$$

(11) $${\mathrm{pH}}_{J}=\log2-\log\;[\sqrt{{\left(2{\beta W}_{J}-K\right)}^{2}+4K\alpha }-\left(2{\beta W}_{J}+K\right)]$$

where α = ma. The continuous line in Figure 10 represents the best interpolation of experimental data when K, α and β are varied. The fit gave a reduced χ-Square value of 0.993. Values of optimized parameters were (1.8 ± 0.2) × 10^−6^ mole·L^−1^, (3.86 ± 0.2) × 10^−4^ mole·L^−1^ and (0.7 ± 0.2) × 10^−5^ mole·L^−1^·g^−1^ for K, α and β, respectively. Based on these results, and considering the presence of the additive evidenced into the cellulose paste, through the analytic determinations discussed above, a reasonable indication concerning the chemical species responsible for the book acidity can be made. This species should have a hydrolysis pK around five. On the other hand, the only freely water-soluble additives were evidenced in the paper alum species. On the contrary, the only iron species seen by PXRD is goethite. This compound cannot contribute to book acidity due to its very low solubility. According to Flynn (1984) [56], the solubility pK_sol_ of this species is, in fact, 41.7, while the required solubility for the relevant acidifying species must be around (3.8 ± 1.3) × 10^−4^ mole·L^−1^ (value of α). On the other hand, Sarpola (2007) [57] indicates a pK equal to 5.52 for the process reported in Equation (12).

(12) $${\mathrm{Al}}^{3+}+H_{2}O\;\to \;\mathrm{Al}{\left(\mathrm{OH}\right)}^{2+}+{\;H}^{+}$$

According to these authors and some of their references [58], the solubility of Al^3+^ and Al(OH)^2+^ are of the order of 10^−4^, in the pH range in book pages, and very close to the value predicted by the discussed model. A final comment can be made with respect to the value of the β factor giving the proportionality between the amount of basic solution absorbed by the book during the nebulization and the local concentration of calcium in the water droplets added to the paper during the pH measurement. A theoretical estimation of the β factor can be made considering that:

1. A 40 μL volume of water droplets is dispersed on the paper to measure the pH, and droplets diffuse on the surface corresponding to the measure electrode surface (0.78 cm^2^);
2. The total surface of the opened pages, hypothesized to be the only absorbers of the nebulized solution, is equal to 1560 cm^2^;
3. The concentration of the Ca^2+^ ion in the nebulized solution is equal to 8.73 × 10^−3^. Simple calculations made on the assumption that the added calcium hydroxide dissolves completely in the water droplet dispersed under the pH measure electrode would lead to a theoretical value of the β factor equal to about 10^−4^; that is to say, a value one order of degree bigger with respect to that furnished by the fit of the titration curve. This apparent discrepancy can be explained, in our opinion, considering that a greater part of the added calcium hydroxide can react with the atmospheric CO_2_, during the drying stage of the paper, becoming calcium carbonate, which remains in the system as an alkaline reserve. On the basis of this hypothesis, the values of β could justify a contribution to the alkaline reserve of the order of about 0.15% of calcium carbonate with respect to the weight of the paper.

### 3.5. Colorimetric and Optical Microscopic Analysis Results

Table 3 reports the values of the chromatic coordinates L*, a* and b*, the total color difference (ΔE*) and the whiteness parameter (W) for both the paper and ink in the four different cases: (a) untreated; (b) deacidified; (c) untreated and aged; (d) deacidified and aged.

It can be seen that the paper, before any treatment, shows strong green and yellow components and has a relatively high lightness (L* = 70.3; a* = −14.2; b* = 16.3). The yellow component is explainable in terms of the cellulose oxidation, which turns the white appearance of paper yellow [59]. The only track that could explain the presence of the green component seems linked to the presence of bunsenite (NiO) in the paper, as evidenced by the PXRD analysis. This mineral is known to occur in dark green crystals and could be present as an impurity in the original material used to prepare the alum. It is worth noticing that the deacidifying treatment does not introduce differences in the colorimetric coordinate of both the paper and the ink, which remain the same as the limit of experimental error. The untreated samples lose luminosity (L*) (more than 10%) and whiteness (W) (around 10%), with a total color difference (ΔE*) of about 11% with aging treatment. Even worse appear to be the aging effects on the ink, which undergoes an increase in luminosity (20%) and whiteness (30%), with a total color difference of 24%. It is not easy to explain in detail the reasons for these variations, but they evidence, as expected, a dramatic effect of the aging on the original untreated book. Instead, the colorimetric coordinates of deacidified and aged samples appear to be quite similar to those of the original book. In particular, the whiteness does not change in the limit of measurement error for both the paper and ink, while the total color difference is almost in the range of the experimental error. These colorimetric findings confirm the protective role of the treatment. The stability of ink with respect to the aerosol treatment is also verified by observing the script characters before and after treatment and before and after aging tests by the optical microscope with a magnification of 1000 times. The total absence of bleeding is thus ascertained.

### 3.6. Cellulose Degree of Polymerization

The degree of polymerization of paper, calculated from the limiting specific viscosities, determined as described above, for different samples of paper (untreated, untreated and aged, deacidified, deacidified and aged) are reported in Table 4. 

Considering that cellulose depolymerization, caused by acid or alkaline hydrolysis and by radical oxidation reactions, appears to be a central mechanism in the aging of paper documents [1,60], the results shown in Table 4 are very encouraging with respect to the goodness of the proposed deacidification mechanism. The aging treatment caused a DP decrease by 55% on the untreated book, while only a 31% DP loss was observed for the deacidified sample. Once more, the reduction of acidity appears to be an important cause of book preservation. The treatment generates a decrease of 15% in DP, probably due to the high pH of the treatment (more than 12). This does not invalidate the aqueous aerosol technique proposed in this paper, as the treatment pH can be modified in the future using alkaline water aerosols obtained by diluting the saturated calcium hydroxide solution or using other types of water-soluble alkaline chemicals. 

### 3.7. Alkaline Reserve Results

The alkaline reserve introduced by the treatment was determined by following the procedure illustrated in Section 2.9. A 19.35 mL volume of NaOH 0.1 N was required to neutralize 20 mL of HCl 0.1N. This corresponds to an alkaline reserve of 0.33%. However, we performed the alkaline reserve test even on the untreated paper. 

This was suggested by the PXRD analysis, which evidenced the presence of calcium carbonate on the untreated sample. In the case of untreated paper, the HCl neutralization required 19.85 mL of NaOH. This means that a small alkaline reserve (0.08%) was already present in the original paper book. Calcium carbonate powder was probably added by the manufacturers of the paper in the past to avoid acidity. Thus, our treatment introduced only about 0.25% of the alkaline reserve. We did not want to push the treatment above pH 6.5 to avoid alkaline hydrolysis problems. On the other hand, the spectroscopic characterization of the paper shows the contemporaneous presence of calcium carbonate and calcium sulfate. The reason why, after more than one hundred years from paper fabrication, calcium carbonate could survive at around pH 4, is probably due to the passivation of this species by a layer of calcium sulfate on the external surface. 

This seems to indicate that the alum rosin was obtained by an alum generated by the reaction of bauxite with sulfuric acid in the presence of carbonate. The presence in the paper of several oxides species such as goethite (FeO(OH)), bunsenite (NiO) and anatase (TiO_2_) indicates that paper cannot contain free sulfuric acid. This is also confirmed by the pK value of the acid species acting into the book obtained by fitting the titration curve of the paper, which is bigger than five, while the pK of sulfuric acid is lower than one.

## 4. Conclusions

This paper presents an experiment of book deacidification, where a nebulized water solution of calcium hydroxide was used. A limited amount of this solution was aspersed on the book, followed by the injection of dry air. In this way, deacidification treatment cycles were performed, maintaining the humidity of the book around 0.2 g of water per page, during the wet phase. The full treatment took about two hours. Only a limited number of pages were kept open during the nebulization treatments to absorb the nebulized solution in order to reduce the time of the experiment, mostly with respect to the different spectroscopic and X-ray diffraction analyses. The book paper additives were characterized by FTIR, PXRD and XRF analyses. This final spectroscopic technique showed that the amount of the added solution during the treatment was almost constant all along the pages of the book kept open. The averaged pH values plotted versus the amount of the weight of the solution absorbed by the book at each of the treatment cycles was interpreted in terms of a simple model, assuming that the book acidity could be attributed to the hydrolysis of an acid cationic species. Taking into consideration the pK and the analytic concentration of the cationic species, obtained by the fit of the titration curve and the spectroscopic and X-ray diffraction data, the cationic species responsible for the book acidity was identified to be the Al^3+^ introduced into the paper with the alum additive. Even if the neutralization of the book was performed up to a pH equal to 6.5, an alkaline reserve of the order of 0.25% was added to the deacidified book. The treatment appeared to preserve the book during aging, as the depolymerization degree (DP) introduced by aging the deacidified paper was found three times smaller with respect to that of the untreated paper. The colorimetric analysis results confirmed the goodness of the treatment with respect to the preservation of the paper and the ink during aging. We are confident with the fact that the deacidification process reported in this paper can result useful in the future to solve such a big problem, in favor of book conservation, considering that: (a) the full time for the deacidification experiment took about two hours, and the treatment of the full amount of the book pages would only provide a longer treatment time (due to the requirement of a longer nebulization time, estimated of the order of 10 h); (b) the treatment can be easily automatized; (c) the humidity introduced into the book during treatment is very limited; (d) the method can be extended, with appropriate apparatus, to a multitude of volumes; (e) the treatment can be generalized by introducing another alkaline medium to the water-based aerosol.

## Figures and Tables

**Table 1:** FTIR wavenumbers, vibrational mode and related chemical compounds (cellulose bands are in blue) (Miller and Wilkins 1952; Garside and Wyeth 2003; Sequeira et al., 2006; Librando et al., 2011).

Wavenumber (cm^−1^)	Functional Chemical Groups	Chemical Compound
3680–3000	OH stretching	Cellulose
2900	CH stretching	Cellulose
2740–2700	CH stretching	Cellulose
2540–2510	CO stretching	CaCO_3_
2230–2130	CO stretching	CaCO_3_
1735	C=O stretching	Cellulose
1650–1635	OH bending/adsorbed water	Cellulose and others
1430	CO stretching	CaCO_3_
1372	CH bending	Cellulose
1340	CH bending	Cellulose
1320	CH bending	Cellulose
1280	CH_2_ twisting	Cellulose
1174	C-C stretching	Cellulose (and others)
1130	SO stretching asym	Ca(SO_4_)·2H_2_O
1090	CO stretching asym	CaCO_3_
1050–1015	SO stretching	Ca(SO_4_)·2H_2_O
870–880	CO stretching asym	CaCO_3_
715	CO stretching asym	CaCO_3_
670	Ca-OH bending	Ca(SO_4_)·2H_2_O
640–610	Ca-OH bending	CaCO_3_
460–430	Ca-OH stretching	CaCO_3_

**Table 2:** Crystalline compound identified in PXRD spectra of the book paper (before and after deacidification).

PXRD Identified Compound	Common Name	2θ (°)	N.° Ref.
KAl(SO_4_)_2_·12H_2_O	K-alum	12.6; 17.9; 20.4; 31.2; 32.3; 34.7; 44.7	R040134
KAl_3_(SO_4_)_2_(OH)_6_	Alunite	17.9; 25.7; 29.6; 39.4; 47.8	R060430
FeO(OH)	Goethite	21.0; 33.6; 34.7; 36.8	R050142
Ca_4_Al_6_O_12_(SO_4_)	Ye’elimite	23.5; 33.6; 36.3; 50.5; 56.6; 58.4; 60.5; 62.4	COD 9009938
TiO_2_	Anatase	25.1; 37.7; 47.8	R060277
CaCO_3_	Calcite	29.6; 39.4; 48.5	R040070
NiO	Bunsenite	37.7; 43.5; 63.5; 75.5	R080121

**Table 3:** Colorimetric coordinates before and after deacidification treatment and before and after aging.

	Colorimetric	Pages Average	SD Pages	Ink Average	SD Ink
	Coordinate	Value		Value	
(a) Untreated	L*	70.4	0.5	51.0	1.0
	a*	−14.2	0.2	3.6	1.0
	b*	16.2	0.2	16.0	0.5
	W	71.8	0.7	45.3	1.2
(b) Deacidified	L*	69.8	0.7	50.7	0.8
	a*	−15.4	0.3	3.0	0.4
	b*	16.6	0.3	16.6	0.6
	W	71.5	1.1	45.1	1.1
Variation between b and a (deacidified − untreated)	ΔL*	−0.6	1.2	−0.3	1.8
	Δa*	−1.2	0.6	−0.6	1.4
	Δb*	0.4	0.5	0.6	1.1
	ΔW	−0.3	1.8	−0.2	2.3
	ΔE*	1.4	0.9	0.9	2.2
(c) Untreated and aged	L*	62.5	0.2	61.7	0.7
	a*	−21.3	0.3	−18.4	1.5
	b*	20.8	0.1	18.5	0.8
	W	64.2	0.5	61.4	1.7
Variation between c and a (Untreated and aged − untreated)	ΔL*	−7.9	0.7	9.8	1.8
	Δa*	−7.1	0.5	−21.9	2.5
	Δb*	4.5	0.2	2.5	1.4
	ΔW	−7.3	1.2	16.1	2.9
	ΔE*	11.5	1.0	24.1	2.6
(d) Deacidified and aged	L*	67.8	0.2	52.9	0.5
	a*	−18.3	0.4	2.7	0.2
	b*	17.5	0.3	18.1	0.5
	W	70.1	0.6	47.7	0.7
Variation between d and a (deacidified and aged − untreated)	ΔL*	−2.6	0.7	1.9	1.5
	Δa*	−2.9	0.7	−0.9	1.2
	Δb*	1.3	0.5	2.1	1.1
	ΔW	−1.7	1.2	2.4	1.9
	ΔE*	4.1	1.0	2.6	2.0

**Table 4:** Paper’s DP before and after deacidification treatment and aging test. The standard deviation on the DP is between 5 and 10%.

Sample	DP
(a) Untreated	825 ± 31
(b) Untreated and aged	369 ± 26
ΔDP = A − B	456 ± 57
Depolymerization (%)	55
(c) Deacidified	700 ± 37
ΔDP = A − C	125 ± 68
Depolymerization (%)	15
(d) Deacidified and aged	568 ± 20
ΔDP = A − D	257 ± 51
Depolymerization (%)	31

## Data Availability

The datasets generated and analyzed during the current study are available from the corresponding author on reasonable request.

## Sample Availability

Samples are not available from the authors.
